# Partitioning the risk of tuberculosis transmission in household contact studies

**DOI:** 10.1371/journal.pone.0223966

**Published:** 2019-10-22

**Authors:** Avery I. McIntosh, Helen E. Jenkins, C. Robert Horsburgh, Edward C. Jones-López, Christopher C. Whalen, Mary Gaeddert, Patricia Marques-Rodrigues, Jerrold J. Ellner, Reynaldo Dietze, Laura F. White

**Affiliations:** 1 Department of Biostatistics, Boston University School of Public Health, Boston, Massachusetts, United States of America; 2 Department of Epidemiology, Boston University School of Public Health, Boston, Massachusetts, United States of America; 3 Section of Infectious Diseases, Department of Medicine, Boston Medical Center and Boston University School of Medicine, Boston, Massachusetts, United States of America; 4 Department of Epidemiology and Biostatistics, College of Public Health, University of Georgia, Athens, Georgia, United States of America; 5 Núcleo de Doenças Infecciosas, Vitória, Brazil; Johns Hopkins University Bloomberg School of Public Health, UNITED STATES

## Abstract

Household contact studies of tuberculosis (TB) are a common way to study disease transmission dynamics. However these studies lack a mechanism for accounting for community transmission, which is known to be significant, particularly in high burden settings. We illustrate a statistical approach for estimating both the correlates with transmission of TB in a household setting and the probability of community transmission using a modified Bayesian mixed-effects model. This is applied to two household contact studies in Vitória, Brazil from 2008–2013 and Kampala, Uganda from 1995–2004 that enrolled households with an individual that was recently diagnosed with pulmonary TB. We estimate the probability of community transmission to be higher in Uganda (ranging from 0.21 to 0.69, depending on HHC age and HIV status of the index case) than in Brazil (ranging from 0.13 for young children to 0.50 in adults). These estimates are consistent with a higher overall burden of disease in Uganda compared to Brazil. Our method also estimates an increasing risk of community-acquired TB with age of the household contact, consistent with existing literature. This approach is a useful way to integrate the role of the community in understanding TB disease transmission dynamics in household contact studies.

## Introduction

Household contact studies for tuberculosis (TB), a common framework for characterizing risk factors for transmission, often involve following cohabitating contacts of an index TB case and testing them for latent tuberculosis infection (LTBI), through a tuberculin skin test (TST) or interferon gamma releasing assay (IGRA). The origin of the infection in co-prevalent LTBI cases (testing positive at study initiation) is challenging to infer, given substantial evidence that infection from a source outside the home (either concurrent or prior to the study) is likely, though not certain, especially in high-prevalence settings.[1–5] Investigators may discard information on co-prevalent infections due to uncertainty in the infection source and only consider those who become infected during follow-up, assuming that these latter incident cases were infected by their diseased household contact. However, substantial information is discarded in this scenario. Further it is not possible to exclude a booster effect of repeated TST rather than a recent M.tb infection particularly in a BCG immunized population. Alternatively, investigators may assume that all observed latent infections were caused by the diseased index case. In either case, information on transmission dynamics is incomplete and unobserved. The omission of what could constitute a substantial pool of study participants or assumption of exclusive within-household transmission can lead to bias in estimates of household risk factors for transmission.[6]

Understanding the source of infection among household contacts has important public health policy implications. Current strategies to reduce the likelihood of progression to TB disease include administering preventive therapy (PT) to household contacts of infectious TB cases, which has been shown to reduce progression to active disease if community-acquired infection rates are low.[7] However this strategy misses many individuals who are not exposed within their household and can be costly in resource-limited settings.[8]

Individual characteristics of a household contact may inform the likelihood of community or household infection. Increased LTBI prevalence by age-stratum has been observed in several populations.[9–12] If LTBI prevalence increases with age, we would expect to observe an increasing probability of community-acquired infection risk with increasing age. It is also feasible that other characteristics of the index case, household contact, and living environment could inform the probability of household transmission.

In what follows, we estimate the probabilities of household and community acquired infection, using data from household contact studies in Vitória, Brazil and Kampala, Uganda. Our approach uses a Bayesian statistical model to infer these probabilities.[6] We show how these probabilities vary by study location, age, and HIV status of the index case.

## Methods

### Patient populations

#### Brazil

Data were taken from the US-Brazil Research Collaboration on Strain Variation in Tuberculosis study, conducted at the Núcleo de Doenças Infecciosas (NDI) in Vitória, Brazil between 2008 and 2013. Investigators enrolled 160 index cases and their 838 household contacts and recorded demographic, household, and index case disease characteristics. Index TB cases were screened and enrolled within 2 weeks after first presenting to their local TB clinic. Acid-Fast Bacilli (AFB) sputum smear, a microscopic examination of stained specimens to detect tuberculosis bacilli, was used to diagnose and grade TB disease; an increasing smear grade indicates a larger number of bacteria per high-powered field on microscopic examination of the sputum. Individuals were only eligible if their AFB smear grade was a 2+ or 3+. No HIV positive index cases were enrolled and HIV testing was not done on household contacts, due to the low prevalence of HIV in this population. Household contacts who were found to have TB disease were not eligible for inclusion in the study. For this study, those household contacts with TB disease diagnosed either within four months prior or three months after the index cases diagnosis were considered co-prevalent cases. Limited information was collected on these individuals. Household contacts of index cases were evaluated with TST for LTBI at screening and again after 8 weeks if the first result was negative. Infected individuals include those who test positive (i.e. induration size of at least 10mm) at either time point. The study protocol and population have been described elsewhere and data are available in S3 File.[13,14]

#### Uganda

This study was performed in Kampala, Uganda between 1995 and 2004 and described in detail elsewhere.[15] Smear positive pulmonary TB cases were enrolled from the Tuberculosis Treatment Center of Mulago Hospital. Their household contacts were enrolled within four weeks of the index case enrollment and followed for two years. TSTs were performed on household contacts at study enrollment and again three months after enrollment. Infected individuals were those who tested positive at either time point (defined as induration size of at least 10mm). Co-prevalent cases were defined as those who were diagnosed with TB disease within three months of the index case. There were 1155 household contacts of 297 infectious TB cases included in this study. Due to the high prevalence of HIV in this population, household contacts of HIV positive (n = 552) and HIV negative (n = 603) index cases were considered separately. Data are available in S4 File.

### Statistical methods

#### The unified probability model

We first estimate the risk of infection from a source outside the household contact study using the Unified Probability Model (UPM), a Bayesian hierarchical model described in detail elsewhere[6] and in the S2 File supplement. In brief, this model partitions the risk of TB infection into two sources: household or “community.” Household transmission is transmission that is attributable to the current index case, whereas so-called community transmission is due to any other source (e.g. outside the household or in a previous time period). Household infection is modeled using a logistic regression formulation, allowing transmission to be dependent on covariates. Community transmission is assumed to be constant in each age group. The joint likelihood of the household and community infection is the product of the household transmission and community transmission models. Bayesian methods are used to estimate posterior distributions (estimates) of the relevant parameters. The probability of household infection is described by

$$\mathrm{logit}(p_{ij}^{H})=X_{ij}\beta +b_{j},$$

where ***X***_*ij*_ describes person and household level covariates, $p_{ij}^{H}$ is the probability that individual *i* in household *j* was infected in the household in the current study, and *b_j_* is the random intercept for household *j*. The probability of community infection is assumed to be constant, and is given by:

$$p^{C}=\frac{exp(\alpha )}{1+exp(\alpha )},$$

where *α* is the log odds of community infection. These probabilities represent the probability of being infected in the community or household for those who are represented by the household contact study. These are combined into a single likelihood given by

$$P(Y_{ij}=1)≝\theta_{ij}=$$

$$p_{ij}^{H}+p^{C}-(p_{ij}^{H}\times p^{C}),$$

where *Y*_*ij*_ is an indicator of the presence of latent infection. We do not comment on the risk of re-infection (or multiple infections) and its role, due to the lack of laboratory methods to detect this and poor understanding of this phenomenon.[16] The outcome variable for each person in this model is positive or negative TST result. The model uses the available covariates to determine the probability of within household transmission versus other transmission in the cross-sectional data.

The UPM outputs two classes of statistics: odds ratios (OR) and credible intervals describing risk factors for household M.tb transmission; and a measure of the probability of acquired infection outside of the current exposure within the household, denoted *p*^*C*^. By partitioning the risk of M.tb infection to allow for more than household acquired infection, the resulting ORs detailing the risk factors for household transmission are estimated with less bias.[6] The UPM accounts for potential correlation between household members via a random effect term, *b_j_* a random intercept included in the logistic portion of the model.

In this study, a common set of covariates is chosen for both countries. Covariates considered include household, host, and index case-level covariates and were considered based on results in the parent studies. In this study, we consider index case (age, gender, chest x-ray results, AFB smear grade), household contact (age, gender, sleeping proximity to index case, smoking status, BCG vaccination), and environmental (number of people per room and presence/absence of co-prevalent disease case(s) in the household) covariates. For the final models, we retained covariates that had p-values less than 0.2 in bivariate analyses for either country. As age of the household contact would have a strong modifying influence on community transmission estimates, we perform the analyses for young children (age less than 5), older children (age 5 to <15), and adults (aged 15+) separately. Additionally, since the presence of co-prevalent cases could have a substantial impact on transmission patterns, we include results from models that adjust for this as a sensitivity analysis in the S1 File supplement. Since limited information was collected on many of these individuals, we only account for their presence or absence in the household.

We show the estimated median of the posterior density for all parameters with their 95% credible intervals. All analyses were performed in R 3.6.0 (r-project.org) using JAGS.[17]

## Results

### TST positivity prevalence

**Fig 1:**
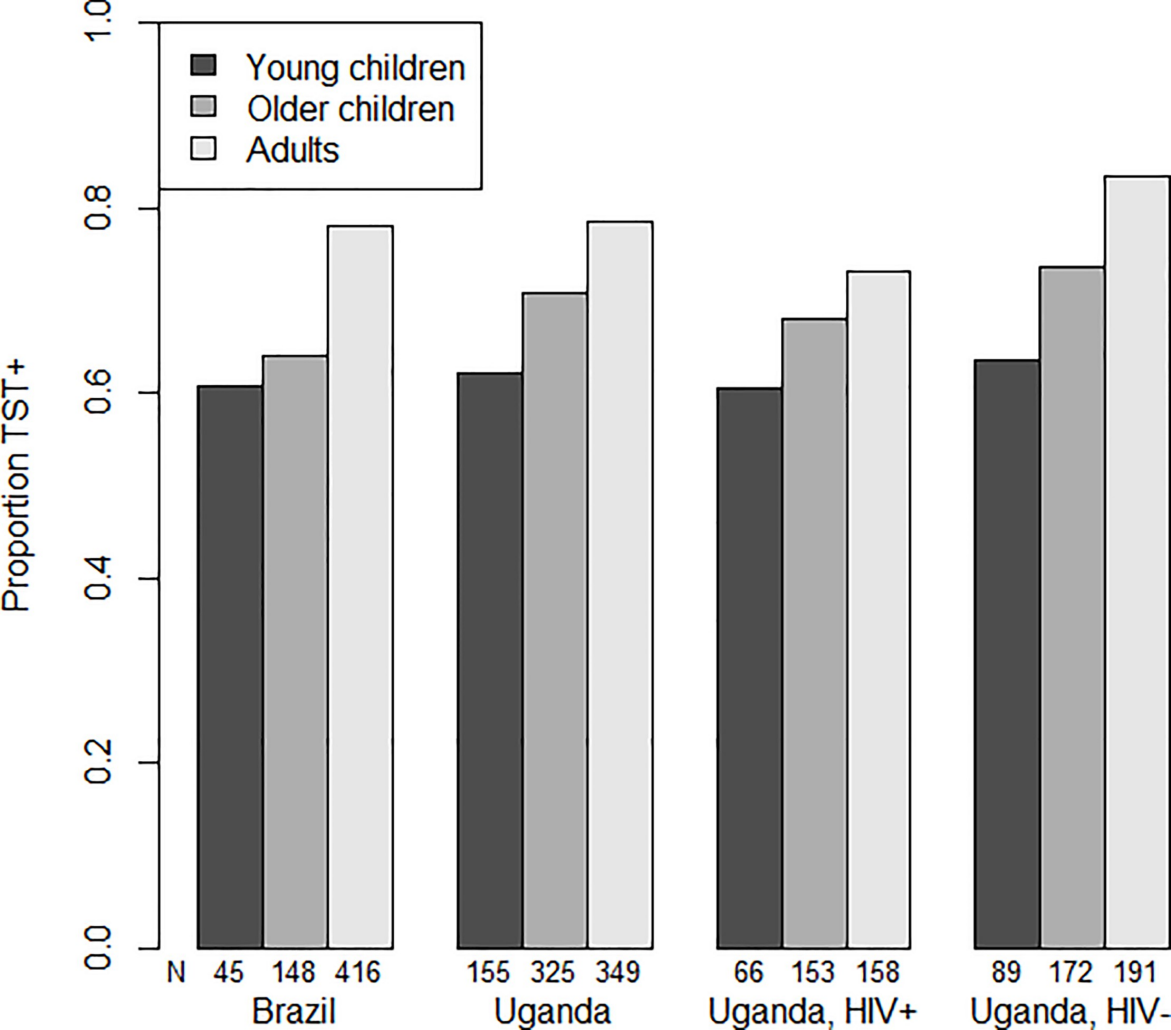
TST prevalence overall and by age group (0–4, 5–14, and 15+) for household contacts in Vitoria Brazil, 2008–2013 and Kampala Uganda, 1995–2004.

Overall individuals with missing data (and excluded from the analysis) did not differ from those included (Table B in S1 File). The TST positivity prevalence among household contacts of an infectious case was lowest among young children and highest among adults (Table 1, Fig 1). Brazilian and Ugandan household contacts had similar LTBI rates. Generally, contacts of HIV- index cases in Uganda had higher rates of TST positivity than contacts of HIV+ index cases.

**Table 1:** Prevalence of latent tuberculosis infection among household contacts in Vitória, Brazil, 2008–2013 and Kampala, Uganda, 1995–2004.

	Age Group		
	Young children (0–4)	Older children (5–14)	Adult (15+)
**Brazil (N = 838)**	45/74 (0.61, 0.49–0.72)	148/231 (0.64, 0.58–0.70)	416/533 (0.78, 0.74–0.82
**Uganda (N = 1153)**	155/249 (0.62, 0.56–0.68)	325/459 (0.71, 0.66–0.75)	349/445 (0.78, 0.74–0.83)
**HIV+ index case (N = 550)**	66/109 (0.61, 0.51–0.70)	153/225 (0.68, 0.61–0.74)	158/216 (0.73, 0.67–0.79)
**HIV- index case (N = 603)**	89/140 (0.64,0.55–0.72)	172/234 (0.74, 0.67–0.79)	191/229 (0.83, 0.78–0.88)

### Probabilities of household and community M.tb infection

The UPM model estimates the probability of community infection to be highest among adult household contacts in all settings, as compared to the other age groups. This probability ranges from 0.11 to 0.50 in Brazil and 0.26 to 0.46 in the Uganda study population, depending on age. Household contacts of HIV- index cases were more likely to be infected in the community compared to household contacts of HIV+ index cases across all age groups (0.40 versus 0.15 for young children; 0.49 versus 0.28 for older children; and 0.64 versus 0.42 for adults). The credible intervals overlapped for all of these groups (Table 2).

**Table 2:** Estimated probability^1^ of household and community transmission and the relative risk of household to community infection. Values in parenthesis are the 95% credible intervals.

	Probability of Household infection	Probability of community infection	RR (p^H^/p^C^)
**BRAZIL**			
**Young children**	0.47 (0.21, 0.64)	0.13 (0.02, 0.38)	3.69 (0.59, 31.78)
**Older children**	0.51 (0.34, 0.63)	0.13 (0.03, 0.28)	3.84 (1.23, 23.09)
**Adults**	0.28 (0.13, 0.65)	0.50 (0.14, 0.64)	0.56 (0.20, 4.71)
**UGANDA, OVERALL**			
**Young children**	0.36 (0.18, 0.56)	0.26 (0.07, 0.44)	1.36 (0.42, 8.47)
**Older children**	0.36 (0.19, 0.59)	0.35 (0.11, 0.51)	1.02 (0.40, 5.12)
**Adults**	0.32 (0.13, 0.67)	0.46 (0.11, 0.65)	0.70 (0.20, 5.98)
**UGANDA, HIV+**			
**Young children**	0.46 (0.22, 0.62)	0.15 (0.02, 0.38)	3.21 (0.57, 26.76)
**Older children**	0.39 (0.19, 0.59)	0.28 (0.08, 0.47)	1.35 (0.42, 7.49)
**Adults**	0.31 (0.10, 0.65)	0.42 (0.09, 0.63)	0.73 (0.16, 7.46)
**UGANDA, HIV-**			
**Young children**	0.23 (0.00, 0.47)	0.40 (0.18, 0.63)	0.57 (0.00, 2.58)
**Older children**	0.24 (0.08, 0.53)	0.49 (0.19, 0.64)	0.48 (0.12, 2.83)
**Adults**	0.19 (0.01, 0.58)	0.64 (0.25, 0.80)	0.30 (0.02, 2.28)

^1^ Results obtained from fitting a multivariate UPM model adjusted for lung cavitations on chest X-ray, AFB smear status, BCG scar, sharing a room with index case and household contact gender.

The relative risk of household to community infection decreased with age, but this decrease was not distinguishable from a relative risk of 1 (Table 2).

Overall the estimated probability of community infection tended to be lower for Brazil (*p*^*C*^ between 0.13 and 0.50) than for Uganda (*p*^*C*^ between 0.26 and 0.46) (Table 2).

### Risk factors for M.tb infection

The study populations were distinct in their demographics and risk factors for infection. On average, both TB index cases and their household contacts were older in Brazil than Uganda (index cases: 35.7 versus 33.0 years; household contacts: 26.1 versus 14.7 years). Index cases in Uganda were more likely to be female than in Brazil (49.0% versus 33.5%), though household contacts had a very similar gender distribution (55.9% versus 56.4% female). Even though index cases with smear grade 1+ were excluded in Brazil, household contacts in both studies appear to have been exposed to index cases with similar levels of advanced disease as smear grade 1+ was uncommon in Uganda. Chest x-ray estimates of the extent of disease were comparable between the two locations, with 44.9% of household contacts in Brazil having an index case with advanced disease compared to 52.7% in Uganda. Ugandan households tended to be more crowded (29.2% with more than three people per room versus 4.4% in Brazil) and household contacts were more likely to share a room with the index case in Uganda than in Brazil (60.8% versus 24.3%) (Table 3). Household contacts of HIV+ and HIV- index cases were similar in Uganda (Table A in S1 File).

**Table 3:** Index and household contact characteristics in Brazil and Ugandan populations. Results are shown overall and by TST status. Statistical significance is determined using logistic regression models fit with GEE.

	Brazil			Uganda		
	Overall (N = 838)	Infected (N = 609)	Not infected (N = 229)	Overall (N = 1153)	Infected (N = 829)	Not infected (N = 324)
*Index Case Characteristics*						
Female	281(33.5)	164(35.4)	77(29.1)	565 (49.0)	401 (48.4)	164 (50.6)
Age, mean (SD)	35.7 (13.4)	35.1 (13.4)	37.1 (13.2)	33.0 (9.0)	32.8 (9.2)	33.4 (8.6)
Karnofsky Score 𢉤 70	42 (5.0)	21 (3.5)	21(9.2)^a^	N/A		
AFB Smear						
1+	N/A	N/A	N/A	86 (7.5)	43 (5.2)	43 (5.2)
2+	168(20.0)	112(18.4)	56(24.5)	143 (12.4)	81 (10.0)	62 (19.1)
3+	670 (80.0)	497(81.6)	173(75.5)^a^	924 (80.1)	705 (85.0)	219 (67.6)^d^
Culture positive	N/A			1131 (98.1)	816 (98.4)	315 (97.2) ^b^
Extent of Disease						
Normal/ Min	49(5.9)	34 (5.6)	15 (6.6)^d^	159 (13.8)	93 (11.2)	66 (20.5)
Moderate	406(49.2)	276 (46.1)	130 (57.3)	385 (33.5)	263 (31.8)	122 (37.9)
Advanced	371(44.9)	289 (48.2)	82 (36.1)	605 (52.7)	471 (57.0)	134 (41.6) ^d^
Missing	12	10	2	3	2	2
Cavitations present	615(74.5)	469 (78.3)	146 (64.3)^d^	633 (54.9)	499 (60.2)	134 (41.4) ^d^
Missing	12	10	2	0	0	0
Co-prevalent case(s)*	53 (6.3)	34 (5.6)	19 (8.3)	161 (14.0)	123 (14.8)	38 (11.7)
*Household Contact Characteristics*						
Age (years)						
Mean(SD)	26.1(19.5)	27.2(19.1)	22.9(20.2)	14.7 (12.9)	15.9 (13.4)	11.7 (11.2)
0–4	74 (8.8)	45 (7.4)	29 (12.7)	249 (21.6)	155 (18.7)	94 (29.0)
5–14	231 (27.6)	148 (24.3)	83 (36.2)	459 (39.8)	325 (39.2)	134 (41.4)
15+	533 (63.6)	416 (68.3)	117 (51.1)^d^	445 (38.6)	349 (42.1)	96 (29.6)^d^
Female	473(56.4)	351(57.6)	122(53.3)^a^	644 (55.9)	471 (56.8)	173 (53.4)
Share room with index case	204 (24.3)	165(27.1)	39 (17.0)^a^	693 (60.8)	504 (61.8)	188 (58.4)
Missing				15	13	2
Smoker	194 (23.3)	159(26.4)	35 (15.3)^a^	68 (5.9)	55 (6.7)	13 (4.0) ^a^
Missing	7	7	0	1	1	0
BCG Scar	686(84.7)	500(84.7)	186(84.5)	845 (73.4)	590 (71.3)	255 (78.7) ^d^
Missing	28	19	9	1	1	0
More than 3 people per room	37 (4.4)	21 (23.5)	16 (7.0)	337 (29.2)	239 (28.8)	98 (30.2)

^a^ 0.10 < p 𢉤 0.20

^b^ 0.05 < p 𢉤 0.10

^c^ 0.01 < p 𢉤 0.05

^d^ p 𢉤 0.01

*77 households had at least one co-prevalent case in Uganda, found of whom had 2 co-prevalence cases.

Presence of cavitations on chest x-ray and AFB smear grade of the index cases, presence of BCG scar, gender of the household contacts, and sharing a room with the index case were included in the UPM multivariable model. In multivariable modeling, cavitation in the index case were associated with LTBI in Uganda for all age groups, but not among those household contacts of HIV negative individuals (Table 4). Results were very similar with the inclusion of an indicator of a co-prevalent case (Tables C and D in S1 File).

**Table 4:** Odds ratios for covariates included in the multivariate models fit with UPM methodology. These models also provide estimates of the community and household infection shown in Table 2.

	Adults			Older children			Younger children		
	Estimate	LCL	UCL	Estimate	LCL	UCL	Estimate	LCL	UCL
BRAZIL									
Cavitations	3.82	0.61	20.9	4.3	0.48	34.66	0.8	0.08	8.28
BCG scar	1.34	0.24	6.73	2.39	0.31	19.74	0.84	0.06	8.65
Smear 3+	1.46	0.20	8.86	1.47	0.18	12.51	1.45	0.15	14.02
Share room with index case	3.04	0.61	17.97	1.96	0.29	13.74	4.86	0.38	49.97
Female gender, HHC	0.91	0.23	3.40	0.96	0.20	4.22	3.46	0.40	26.76
UGANDA, OVERALL									
Cavitations	3.42	1.29	17.63	6.34	1.52	40.64	6.63	1.25	38.83
BCG scar	0.51	0.12	1.23	0.37	0.07	1.16	0.82	0.12	4.42
Smear 2+	0.82	0.09	4.85	0.66	0.06	4.45	0.39	0.03	3.45
Smear 3+	5.03	1.12	24.85	4.32	0.71	22.12	2.1	0.32	16.34
Share room with index case	2.24	0.8	9.74	1.23	0.34	4.56	1.33	0.2	7.42
Female gender	1.06	0.35	4.12	1.05	0.34	3.19	1.08	0.21	6.02
UGANDA, HIV+									
Cavitations	5.64	1.08	36.1	9.78	1.96	58.51	10.13	1.37	76.34
BCG scar	0.58	0.1	2.48	0.62	0.11	2.44	0.54	0.08	3.65
Smear 2+	0.77	0.07	4.86	0.34	0.04	2.1	0.46	0.04	3.7
Smear 3+	3.14	0.43	23.68	3.46	0.59	20.86	3.5	0.45	29.8
Share room with index case	1.6	0.33	8.18	0.48	0.11	2.5	1.44	0.17	11.69
Female gender	0.5	0.08	2.75	0.86	0.22	2.77	0.48	0.07	2.85
UGANDA, HIV-									
Cavitations	1.17	0.15	8.06	1.74	0.2	14.39	1.58	0.12	13.61
BCG scar	0.42	0.06	2.58	0.33	0.04	3.41	1.04	0.08	10.34
Smear 2+	0.64	0.06	7.17	1.57	0.11	19.06	0.71	0.06	8.55
Smear 3+	2.28	0.2	20.72	1.25	0.1	11.74	0.67	0.06	6.97
Share room with index case	3.13	0.24	23.41	2.53	0.34	23.42	0.8	0.07	8.34
Female gender	2.5	0.29	21.55	1.46	0.21	10.51	1.57	0.13	14.83

## Discussion

This study is noteworthy in that we estimate both the probability of community- and household-acquired *M*. *tuberculosis* infection, and individual-level risk factors of infection from household contact data, while controlling for confounding and accounting for household clustering effects. This analytic method allows for infections to be acquired outside the context of the current household contact study (“community” transmission) using a Bayesian generalized linear mixed effects model. We estimate odds ratios describing the association between TST positivity in household contacts and index case, household contact and environmental factors. We apply this method to data from household contact studies in Vitória, Brazil and Kampala, Uganda stratified by household contact age group. This approach can be used for any household contact study to both create less biased estimates of the association between covariates and LTBI[6] and to estimate the risk of community and household transmission of TB.

Our relative estimates of community transmission between Brazil and Uganda are consistent with WHO estimates of TB disease incidence. Brazil’s 2016 estimated TB incidence rate was 42 (36–48) per 100,000 while Uganda’s was considerably higher at 201 (118–306) per 100,000. Consistent with this, we estimate transmission from sources other than the index case to be more likely in Uganda than Brazil across all age groups.

This association between community transmission and prevalence of TB is consistent with published evidence, indicating that the risk of exposure from sources other than the index case in a household is more common in areas where TB disease is highly prevalent. Several studies using restriction fragment length polymorphism (RFLP) analysis in the Western Cape Province of South Africa where there is high TB prevalence, found evidence supporting substantial community transmission. In one study, at most 19% of household contacts could have been infected within their household using RFLP analysis[2] whereas another study found that 55% of household contacts had different strains than those of infectious household members, implying that no more than 45% were true infector-infectee pairs.[18] In England, where TB prevalence is low, only 7.7% of all reported cases lived with another TB case, which could be due to a substantial percentage of M.tb infections being acquired outside the household or in a higher prevalence country prior to immigration.[19,20] Among those within the same household, 64% were confirmed genetically as linked cases with another 11% being probable[21] consistent with the finding that lower community prevalence is associated with higher within household transmission. It is important to note that patterns in the linkages observed between active cases of disease may not mirror those observed in infections.

We also show that children are more likely to be infected in the household than adults as observed in other studies that have shown that children are most likely to be infected by an adult, particularly an adult within their household, than by a community source.[22] However, even this risk of within-household transmission to children might be low[7,23] which is consistent with our estimates of the probability of household infection ranging from 0.23 among contacts of HIV- TB cases in Uganda to 0.47 in Brazil. This low level of household transmission in children is consistent with a study of child contacts of TB cases in Cape Town, South Africa where two of the six children with TB disease and RFLP analysis performed had strains that matched the infectious adult TB case in their household. However, information on other household exposures were not collected and small numbers make it challenging to generalize these findings.[24] Other studies, notably in Uganda, have shown mismatches in strains.[15,25] Recent evidence using whole genome sequencing in Canada suggests that pediatric TB is often acquired in the country of origin for children that are foreign born, or by travel to another country for those whose parents were foreign born. Among Canadian-born children infection was most commonly acquired in Canada, with transmission most frequently occurring from visitors to the home or a household member (foreign born other Canadian born) supporting the hypothesis that children in low prevalence settings are primarily exposed in the home.[21]

Despite early uncertainty, a meta-analysis of the relative transmissibility of HIV+ and HIV- individuals with TB disease showed that rates of LTBI were similar regardless of the HIV status of the index case.[26] More recent studies have suggested that individuals with TB disease who are HIV+ tend to transmit disease less frequently than HIV- individuals[27,28] which is supported by our observation of a slightly lower prevalence of infection among household contacts of HIV+ index cases. However, overall we estimate household transmission of *M*. *tuberculosis* to be higher for household contacts of HIV+ index cases than for those with an HIV- index case. This would imply that HIV+ cases might in fact be more infectious to their immediate contacts and those contacts are slightly less likely to be infected with TB in the community. This could be due to more than biological phenomena, but also might be attributable to unique patterns of behavior and environmental conditions of these households.

There are several potential limitations of our approach. The probability of community-acquired infection is assumed to be constant for all persons, and not informed by factors that could also lead to within-household transmission. This assumption is obviously a simplification and we show here that stratification by age group is important. Further statistical development to allow this to vary by additional factors would potentially improve estimation and provide further insight on the transmission dynamics. However additional data, such as that provided by having community controls, would likely be needed. Additionally, we were limited in our comparison of these two geographical regions by differences between the data collected in each study. For instance, the Brazilian study only enrolled index cases with a smear grade of at least 2+ while the Ugandan study enrolled all smear positive cases. However, the severity of disease, as measured on chest x-ray did not indicate that Brazilian cases were more severe and there were few smear 1+ in Uganda. Other factors such as occupation, presence of other active cases in the household, duration of infectiousness, and contact patterns with other TB cases would also potentially be important to include, but were not consistently collected and/or cannot be accurately ascertained retrospectively. Additionally, there could be potential misclassification of young children who were BCG vaccinated and thus might be TST+ when they are in fact uninfected. Finally, we are limited in the data available to assess transmission risks. It is possible that there are other unmeasured factors that strongly correlate with transmission, such as reinfection and cough aerosols, that were not assessed because they were not a part of these studies or are unobservable. As with all studies this has the potential to introduce bias that is not possible to assess.

We note the limitation in interpreting the estimated probability of community-acquired infection. This value is interpretable only for the household contacts enrolled in the study and is not a broad community-level parameter. This is because households enrolled in these studies are not a random sample of the larger population, but meet strict criteria (e.g. presence of a TB case individual, appropriate household size and composition, and willingness to consent to study participation). Therefore, the probability of community-acquired infection estimated here is not easily generalizable to others in the community. Additionally, it is possible that those who live with someone with TB might have a higher risk of M.tb infection because they share other risk factors, independent of transmission. It is possible that with the inclusion of community controls and additional information on the community, one could derive more precise community level estimates of the probability of household and community acquired transmission.

We also note that we do not specifically estimate or account for reinfection. Individuals who are TST positive prior to exposure in the current household contact study could be re-infected and if their covariate patterns line up with household exposure, they would contribute to that probability. This model does not disentangle that dynamic, which is clearly important, but unmeasurable with current diagnostics.

We show that household contact studies can be helpful in inferring community transmission dynamics among household contacts of an infectious TB case by using a method that estimates both community and household M.tb transmission probabilities. We show that the likelihood of transmission from sources other than the index case in the household increases with age and varies consistent with the background burden of disease in a community (as measured by national TB incidence rates).

## Supporting information

S1 File Supplementary table. Table A. Index and household contact characteristics by HIV status in Uganda. Table B. Comparison between those with and without missing data. Table C. Estimates with inclusion of information about co-prevalent cases. Table D. Odds ratios from models with information on co-prevalent cases included.
(DOCX)

S2 File Additional information on the unified probability model. (DOCX)

S3 File Brazilian household contact study data. (XLSX)

S4 File Uganda household contact study data. (XLSX)
