# Supplementary material for: Partitioning the risk of tuberculosis transmission in household contact studies
Source: PLoS One. 2019 Oct 22;14(10):e0223966. doi: 10.1371/journal.pone.0223966 (PMC6804987; doi:10.1371/journal.pone.0223966)
Supplement: S1 File — Table A. Index and household contact characteristics by HIV status in Uganda. Table B. Comparison between those with and without missing data. Table C. Estimates with inclusion of information about co-prevalent cases. Table D. Odds ratios from models with information on co-prevalent cases included. (DOCX) [file pone.0223966.s001.docx]

**SUPPLEMENTARY TABLES**

**Table A.** Index and Household contact characteristics of the contacts of HIV positive and HIV negative index cases in Uganda. Results are shown overall and by TST infection status. Statistical significant is determined using logistic regression models fit with GEE.

|  | Uganda HIV+ Index cases | | | Uganda HIV- index cases | | |
| --- | --- | --- | --- | --- | --- | --- |
|  | Overall  (N=550) | Infected  (N=377) | Not infected  (N=173) | Overall (N=603) | Infected (N=452) | Not infected (N=151) |
| *Index Case Characteristics* | | | | | | |
| Female | 262 (47.6) | 185 (49.1) | 77 (44.5) | 303 (50.2) | 216 (47.8) | 87 (57.6) ^c^ |
| Age, mean (SD)  missing | 34.4 (7.6) | 34.3 (8.0) | 34.7 (6.8) | 31.6 (10.0) | 31.6 (9.9) | 31.9 (10.2) |
| AFB Smear  1+  2+  3+ | 54 (9.8)  103 (18.7)  393 (71.5) | 27 (7.2)  52 (13.8)  298 (79.0) | 27 (15.6)  51 (29.5)  95 (54.9) ^d^ | 32 (5.3)  40 (6.6)  531 (88.1) | 16 (3.5)  29 (6.4)  407 (90.0) | 16 (10.6)  11 (7.3)  124 (82.1)^c^ |
| Culture positive | 533 (96.9) | 366 (97.1) | 167 (96.5) ^b^ | 598 (99.2) | 450 (99.6) | 148 (98.0) ^b^ |
| Extent of Disease  Normal/  Min  Moderate  Advanced  Missing | 86 (15.6)  203 (36.9)  261 (47.5)  0 | 40 (10.6)  137 (36.3)  200 (53.1)  0 | 46 (26.6)  66 (38.2)  61 (35.3) ^d^  0 | 73 (12.2)  182 (30.4)  344 (57.4)  4 | 53 (11.8)  126 (28.0)  271 (60.2)  2 | 20 (13.4)  56 (37.6)  73 (49.0)  2 |
| Cavitations present  Missing  Coprevalent Case(s) | 248 (45.1)  0  81 (14.7) | 207 (54.9)  0  62 (15.4) | 41 (23.7) ^d^  0  19 (11.0) | 385 (63.8)  0  80 (13.3) | 292 (64.6)  0  61 (13.5) | 93 (61.6)  0  19 (12.6) |
| *Household Contact Characteristics* | | | | | | |
| Age (years)  Mean(SD)  0-5  6-14  15+ | 14.8 (12.7)  109 (19.8)  225 (40.9)  216 (39.3) | 16.0 (13.4)  66 (17.5)  153 (40.6)  158 (41.9) | 12.2 (10.7)  43 (24.9)  72 (41.6)  58 (33.5)^a^ | 14.7 (13.1)  140 (23.2)  234 (38.8)  229 (38.0) | 15.9 (13.4)  89 (19.7)  172 (38.1)  191 (42.3) | 11.1 (11.7)  51 (33.8)  62 (41.1)  38 (25.2) ^d^ |
| Female | 310 (56.4) | 208 (55.2) | 102 (59.0) | 334 (55.4) | 263 (58.2) | 71 (47.0) ^c^ |
| Share room with index case  missing | 336 (62.2)  10 | 228 (61.8)  8 | 108 (63.2)  2 | 356 (59.5)  5 | 276 (61.7)  5 | 80 (53.0)  0 |
| Smoker  missing | 35 (6.4)  1 | 26 (6.9)  1 | 9 (5.2)  0 | 33 (5.5)  0 | 29 (6.4)  0 | 4 (2.7) ^a^  0 |
| BCG Scar  missing | 402 (73.2)  1 | 267 (71.0)  1 | 135 (78.0) ^b^  0 | 443 (73.5)  0 | 323 (71.5)  0 | 120 (79.5) ^c^  0 |
| More than 3 people per room | 151 (27.5) | 100 (26.5) | 51 (29.5) | 186 (30.8) | 139 (30.8) | 47 (31.1) |

^a^ 0.10 < p < 0.20

^b^ 0.05 < p < 0.10

^c^ 0.01 < p < 0.05

^d^ p < 0.01

**Table B. Comparison between those with missing data and those including in final models.**

|  | Brazil | | Uganda | |
| --- | --- | --- | --- | --- |
|  | Complete data  (N=799) | Missing  (N=39) | Complete data  (N=1137) | Missing  (N=18) |
| *Index Case Characteristics* | | | | |
| Female | 265 (33.2) | 16 (41.0) | 560 (49.3) | 5 (27.8) |
| Age, mean (SD) | 35.4 (13.3) | 40.3 (14.6) | 32.9 (9.0) | 36.6 (9.3) |
| AFB Smear  1+  2+  3+ | N/A  157 (19.6)  642 (80.4) | N/A  11 (28.2)  28 (71.8) | 86 (7.6)  139 (12.2)  912 (80.2) | 0  4 (22.2)  14 (77.8) |
| *Household Contact Characteristics* | | | | |
| Age  0-4  5-14  15+ | 73 (9.1)  228 (28.5)  498 (62.3) | 1 (2.6)  3 (7.7)  35 (89.7) | 248 (21.8)  450 (39.6)  439 (38.6) | 3 (16.7)  9 (50.0)  6 (33.3) |
| Female | 450 (56.3) | 23 (59.0) | 633 (44.3) | 13 (72.2) |
| Share room with index case | 195 (24.4) | 9 (23.1) | 691 (60.8) | 2 (66.7) |
| More than 3 people per room | 34 (4.3) | 3 (7.7) | 333 (29.3) | 4 (22.2) |

**Table C.** Estimated probability^1^ of household and community transmission and the relative risk of household to community infection including information on the presence/absence of co-prevalent cases in the model. Values in parenthesis are the 95% credible intervals.

|  | Probability of Household infection | Probability of community infection | RR (p^H^/p^C^) |
| --- | --- | --- | --- |
| BRAZIL | | | |
| Young children | 0.49 (0.21, 0.65) | 0.13 (0.02, 0.39) | 3.85 (0.57, 34.51) |
| Older children | 0.51 (0.34, 0.63) | 0.13 (0.03, 0.28) | 3.84 (1.23, 23.09) |
| Adults | 0.27 (0.13, 0.65) | 0.51 (0.14, 0.65) | 0.52 (0.20, 4.64) |
| UGANDA, OVERALL | | | |
| Young children | 0.35 (0.16, 0.56) | 0.28 (0.07, 0.46) | 1.26 (0.37, 7.59) |
| Older children | 0.33 (0.18, 0.57) | 0.37 (0.14, 0.52) | 0.88 (0.35, 4.28) |
| Adults | 0.32 (0.12, 0.67) | 0.46 (0.11, 0.65) | 0.70 (0.19, 5.77) |
| UGANDA, HIV+ | | | |
| Young children | 0.46 (0.19, 0.62) | 0.15 (0.02, 0.39) | 3.21 (0.51, 26.16) |
| Older children | 0.37 (0.19, 0.59) | 0.30 (0.09, 0.47) | 1.26 (0.40, 6.85) |
| Adults | 0.29 (0.08, 0.63) | 0.45 (0.11, 0.64) | 0.65 (0.13, 5.92) |
| UGANDA, HIV- | | | |
| Young children | 0.23 (0.01, 0.48) | 0.41 (0.17, 0.62) | 0.57 (0.01, 2.97) |
| Older children | 0.23 (0.07, 0.50) | 0.50 (0.24, 0.65) | 0.46 (0.11, 2.07) |
| Adults | 0.20 (0.03, 0.66) | 0.62 (0.18, 0.80) | 0.33 (0.03, 3.75) |

^1^ Results obtained from fitting a multivariate UPM model adjusted for lung cavitations on chest X-ray, AFB smear status, BCG scar, sharing a room with index case, household contact gender, and presence of co-prevalent case(s) in household.

**Table D.** Odds ratios for covariates included in the multivariate models fit with UPM methodology including the presence/absence of co-prevalent cases in the model. These models also provide estimates of the community and household infection shown in Table 2.

|  | Adults | | | Older children | | | Younger children | | |
| --- | --- | --- | --- | --- | --- | --- | --- | --- | --- |
|  | Estimate | LCL | UCL | Estimate | LCL | UCL | Estimate | LCL | UCL |
| BRAZIL | | | | | | | | | |
| Cavitations | 3.96 | 0.59 | 27.97 | 4.38 | 0.55 | 36.05 | 0.8 | 0.08 | 8.49 |
| BCG scar | 1.38 | 0.23 | 7.03 | 2.27 | 0.25 | 17.87 | 0.86 | 0.08 | 10.48 |
| Smear 3+ | 1.42 | 0.19 | 9.38 | 1.55 | 0.2 | 11.5 | 1.52 | 0.17 | 14.4 |
| Share room with index case | 3.19 | 0.61 | 21.72 | 2.05 | 0.3 | 13.9 | 4.38 | 0.37 | 41.54 |
| Female gender, HHC | 0.91 | 0.19 | 3.33 | 1.01 | 0.23 | 4.36 | 3.31 | 0.41 | 32.12 |
| Co-prev case(s) | 0.64 | 0.06 | 8.5 | 0.95 | 0.08 | 12.28 | 1.24 | 0.08 | 15.3 |
| UGANDA, OVERALL | | | | | | | | | |
| Cavitations | 3.2 | 1.24 | 17.74 | 7.11 | 1.6 | 46.7 | 6.96 | 1.15 | 42.45 |
| BCG scar | 0.47 | 0.1 | 1.21 | 0.34 | 0.06 | 1.21 | 0.81 | 0.11 | 4.68 |
| Smear 2+ | 0.77 | 0.08 | 4.43 | 0.62 | 0.06 | 4.99 | 0.37 | 0.04 | 3.69 |
| Smear 3+ | 4.7 | 1.03 | 25.58 | 4.09 | 0.67 | 26.17 | 1.99 | 0.27 | 17.07 |
| Share room with index case | 2.24 | 0.75 | 9.66 | 1.25 | 0.34 | 4.81 | 1.27 | 0.16 | 8.14 |
| Female gender | 1.04 | 0.35 | 3.81 | 1.06 | 0.3 | 3.24 | 1.09 | 0.21 | 5.7 |
| Co-prev case(s) | 2.54 | 0.49 | 12.49 | 0.97 | 0.16 | 7.09 | 0.77 | 0.09 | 6.41 |
| UGANDA, HIV+ | | | | | | | | | |
| Cavitations | 5.45 | 0.9 | 37.17 | 10.55 | 2.08 | 58.92 | 10.27 | 1.22 | 70.16 |
| BCG scar | 0.57 | 0.08 | 2.59 | 0.63 | 0.11 | 2.66 | 0.51 | 0.07 | 3.39 |
| Smear 2+ | 0.73 | 0.06 | 5.62 | 0.31 | 0.04 | 2.3 | 0.41 | 0.04 | 4.27 |
| Smear 3+ | 3.2 | 0.36 | 22.33 | 3.14 | 0.59 | 23.74 | 3.52 | 0.49 | 27.07 |
| Share room with index case | 1.64 | 0.28 | 7.76 | 0.47 | 0.09 | 2.22 | 1.54 | 0.21 | 9.92 |
| Female gender | 0.49 | 0.08 | 3.18 | 0.82 | 0.17 | 2.98 | 0.48 | 0.08 | 2.96 |
| Co-prev case(s) | 1.04 | 0.14 | 7.36 | 0.77 | 0.11 | 5.31 | 1.76 | 0.16 | 18.69 |
| UGANDA, HIV- | | | | | | | | | |
| Cavitations | 1.17 | 0.14 | 7.70 | 1.76 | 0.18 | 16.72 | 1.60 | 0.13 | 16.20 |
| BCG scar | 0.45 | 0.05 | 2.68 | 0.33 | 0.04 | 3.83 | 1.14 | 0.09 | 10.53 |
| Smear 2+ | 0.56 | 0.05 | 5.80 | 1.63 | 0.11 | 22.75 | 0.72 | 0.05 | 9.47 |
| Smear 3+ | 2.40 | 0.20 | 17.17 | 1.25 | 0.09 | 11.02 | 0.72 | 0.07 | 6.04 |
| Share room with index case | 2.95 | 0.35 | 21.97 | 2.46 | 0.25 | 24.04 | 0.85 | 0.07 | 8.00 |
| Female gender | 2.47 | 0.31 | 18.88 | 1.44 | 0.17 | 9.46 | 1.62 | 0.17 | 19.81 |
| Co-prev case(s) | 3.33 | 0.23 | 30.39 | 0.99 | 0.08 | 10.00 | 0.42 | 0.03 | 5.60 |
