# Supplementary material for: Partitioning the risk of tuberculosis transmission in household contact studies
Source: PLoS One. 2019 Oct 22;14(10):e0223966. doi: 10.1371/journal.pone.0223966 (PMC6804987; doi:10.1371/journal.pone.0223966)
Supplement: S2 File — (DOCX) [file pone.0223966.s002.docx]

**Supplemental information**

**The unified probability model**

The partition of risk of TB infection from a household or community source is accomplished by the use of a Bayesian hierarchical model, which we refer to as the Unified Probability Model (UPM). The model takes as input an outcome variable for each person (positive or negative TST result), an indicator of household membership, and any transmission predictor variables of interest to the investigators. These variables can be individual predictors, such as smoking status or sex, or household-constant variables, such as index case disease characteristics, or number of windows in the home. The model outputs two classes of statistics: odds ratios (OR) and credible intervals describing risk factors for household TB transmission; and a measure of the probability of community-acquired infection in a population with a cohabitating infectious TB case, *p^C^*.

For this analysis, we specify non-informative flat prior probability distributions on predictors of household transmission and the probability of community-acquired transmission. In McIntosh, et al., the UPM framework is shown to be robust to the prior probability distribution of the probability of community infection, and even for overt prior misspecification the model accurately estimates both community infection risk, and individual- and household-level risk factors.

The 5% cutpoint is used for all intervals and error bars, corresponding to 95% credible intervals (CI) for all posterior median estimates.

We apply the UPM as described in McIntosh, et al.(6), however, here we append the UPM framework to additionally produce a measure of the probability of household-acquired TB infection in a population with a cohabitating TB case, *p^HH^*. All statistical analysis is performed in the software package R, version 3.3.1, using package *upmfit*.(28,29)

To derive a posterior probability distribution and credible interval for the probability of household-acquired infection, we first analyzed the probability a person is TST positive by including a term, *p^TB^*, into the posterior distribution sampler for the UPM. The *p^TB^* term is given a prior probability distribution, and together with the observed stratum-specific proportion of persons found to be TST positive, can yield a posterior probability distribution that a person is TST positive. Samples from this distribution can then be combined with the posterior *p^C^* distribution samples generated through the UPM to yield a distribution for the posterior probability a person is infected from the household.

The prior probability distribution for the proportion of persons testing positive for TB is specified for each age-stratified subpopulation as relatively non-informative: a Beta-distributed random variable with primary and secondary shape hyperparameters both equal to 1.2. This prior specification creates a nearly uniform prior distribution on the unit interval, with the density tapering at the extremes of 0 and 1. Other prior probability distributions could be plausibly specified to reflect *a priori* beliefs about the rate of TB infection, such as a prior distribution informed by previous prevalence observations, or a transformation-invariant benchmarking Jeffry’s prior. However, for these populations the posterior distribution for *p^TB^* is barely influenced by prior specification, as the posterior distribution is here informed by the substantial sample size in each subpopulation. Sensitivity analysis on posterior distributions of *p^TB^* using alternative prior specifications yielded changes in posterior quantiles generally only at the thousandths decimal place.

We define the model for *p^TB^* and *p^HH^* as follows: for given population *V* in set {Brazil, Uganda}, population-specific age stratum *a_V_*, with *k_V_* a being the number of TST positive cases in population *V* and age stratum *a*, *n_V_* a being the population- specific stratum sample size, *p^TB^* being the probability of TB infection for the given population, and the age- and population-specific proportion of TST positive persons denoted *D* = *k_Va_*/*n_Va_*, we define prior probability

$$p^{TB}\sim\mathrm{Beta}\left( 1.2, 1.2 \right),$$

with likelihood

$$D|p^{TB}\sim\mathrm{Bern}\left( p^{TB} \right),$$

which by prior conjugacy to the binomial likelihood, the so-called Beta-Binomial distribution, implies that the posterior distribution of *p^TB^* is also distributed Beta:

$$p^{TB}|D \sim Beta(1.2+k_{Va}, 1.2+n_{Va}-k_{Va})$$

For brevity we henceforth omit the conditionals in the notation for the posterior probability distributions of *p^TB^*|*D* to read as simply *p^TB^*.

One can then derive an estimator for household-acquired infection for a given population *V* and age-stratum *a_V_* by subtraction of the posterior median probability of community- acquired infection and the converse of posterior median household-acquired infection parameter from 1. Note that for Binomial Bayes estimation there is a closed-form posterior mean estimator, which here would be (1.2+k_Va)⁄(2.4+n_Va ). However, we feel the posterior median estimate derived from our MCMC sampler is a more robust estimator. The sampler performs well for all subpopulations analyzed, indicating posterior median convergence. Furthermore, if the posterior distribution is roughly Gaussian, the mean and median will coincide, while if the posterior distribution is non-normal, the median will better represent the posterior distribution centrality.

The posterior probability distribution of household-acquired infection will be:

$$p^{HH}=1-\left( p^{C}+\left( 1-p^{TB} \right) \right).$$

That is, since (1− *p^TB^*) + *p^HH^* + *p^C^* = 1, the posterior probability of household infection for age stratum *a_V_* will be 1 minus the probability of community-acquired infection minus the probability of no infection at all. At each iteration of the MCMC sampler, a sample from the posterior distribution of *p^C^* is paired with a posterior sample of *p^TB^* and input into the above formula do derive a sample of the posterior distribution of *p^HH^*. The final outputs of the model are exponentiated model coefficients for household-acquired risk, and terms *p^HH^* and *p^C^*.
